# Stomatal Closure and SA-, JA/ET-Signaling Pathways Are Essential for *Bacillus amyloliquefaciens* FZB42 to Restrict Leaf Disease Caused by *Phytophthora nicotianae* in *Nicotiana benthamiana*

**DOI:** 10.3389/fmicb.2018.00847

**Published:** 2018-04-27

**Authors:** Liming Wu, Ziyang Huang, Xi Li, Liumin Ma, Qin Gu, Huijun Wu, Jia Liu, Rainer Borriss, Zhen Wu, Xuewen Gao

**Affiliations:** ^1^College of Plant Protection, Nanjing Agricultural University, Nanjing, China; ^2^Key Laboratory of Monitoring and Management of Crop Diseases and Pest Insects, Ministry of Education, Nanjing, China; ^3^Chongqing Key Laboratory of Economic Plant Biotechnology, College of Forestry & Life Science, Chongqing University of Arts and Sciences, Yongchuan, China; ^4^Nord Reet UG, Greifswald, Germany; ^5^Fachgebiet Phytomedizin, Institut für Agrar-und Gartenbauwissenschaften, Humboldt-Universität zu Berlin, Berlin, Germany; ^6^College of Horticulture, Nanjing Agricultural University, Nanjing, China

**Keywords:** *Bacillus amyloliquefaciens* FZB42, stomata, ABA, ISR, *Nicotiana benthamiana*

## Abstract

*Bacillus amyloliquefaciens* FZB42 is a plant growth-promoting rhizobacterium that induces resistance to a broad spectrum of pathogens. This study analyzed the mechanism by which FZB42 restricts leaf disease caused by *Phytophthora nicotianae* in *Nicotiana benthamiana*. The oomycete foliar pathogen *P. nicotianae* is able to reopen stomata which had been closed by the plant innate immune response to initiate penetration and infection. Here, we showed that root colonization by *B. amyloliquefaciens* FZB42 restricted pathogen-mediated stomatal reopening in *N. benthamiana*. Abscisic acid (ABA) and salicylic acid (SA)-regulated pathways mediated FZB42-induced stomatal closure after pathogen infection. Moreover, the defense-related genes *PR-1a*, *LOX*, and *ERF1*, involved in the SA and jasmonic acid (JA)/ethylene (ET) signaling pathways, respectively, were overexpressed, and levels of the hormones SA, JA, and ET increased in the leaves of *B. amyloliquefaciens* FZB42-treated wild type plants. Disruption of one of these three pathways in *N. benthamiana* plants increased susceptibility to the pathogen. These suggest that SA- and JA/ET-dependent signaling pathways were important in plant defenses against the pathogen. Our data thus explain a biocontrol mechanism of soil rhizobacteria in a plant.

## Introduction

Plants have evolved a variety of inducible defense mechanisms to protect themselves against pathogen attack. Well-studied, classic examples of induced resistance are: (1) the activation of systemic acquired resistance (SAR), triggered by infection with necrosis-inducing pathogens (Van Wees et al., 2008); and (2) rhizobacteria-induced systemic resistance (ISR), triggered by some non-pathogenic rhizobacteria (Van Loon et al., 1998; Hammerschmidt, 1999) such as plant growth-promoting rhizobacteria (PGPR) (Conrath et al., 2002).

The plant hormones jasmonic acid (JA), salicylic acid (SA), and ethylene (ET) are important signaling molecules in SAR and ISR (Glazebrook, 2001; Pieterse et al., 2014). SAR involves the SA-dependent signaling pathway. Initiation of SAR involves local and systemic increases in endogenously synthesized SA, which causes activation of the regulatory protein NPR1 and then NPR1-dependent expression of genes that encode pathogenesis-related (PR) proteins including *PR-1a* (Ward et al., 1991; Niu et al., 2011). Transgenic *A. thaliana* NahG plants expressing the bacterial *nahG* gene, which encodes the SA-degrading enzyme salicylate hydroxylase, are compromised in *PR* gene expression and SAR (Gaffney et al., 1993). In contrast, ISR requires the JA- and ET-pathways, and is associated with expression of defensin 1.2 (Pieterse et al., 1998; Van Oosten et al., 2008). However, dependence on both SA- and JA/ET-signaling pathways is also observed. For example, colonization of *Arabidopsis* roots by *Trichoderma atroviride* IMI 206040 trigger ISR by inducing the expression of SA and JA/ET pathways simultaneously to confer resistance against hemibiotrophic and necrotrophic phytopathogens (Salas-Marina et al., 2011).

Plant growth-promoting rhizobacteria-mediated ISR, induced by *Bacillus* spp. for example, has been demonstrated in many plant species including melon, bean, tomato, tobacco, and the model plant *A. thaliana* (Van Loon et al., 1998). García-Gutiérrez et al. (2013) demonstrated that *B. subtilis* UMAF6639 confers protection to melon plants against cucurbit powdery mildew by activation of JA- and SA-dependent defense responses. *B. cereus* AR156 has been shown to trigger ISR in *A. thaliana* by simultaneously activating the SA and JA/ET signaling pathways against *Pseudomonas syringae* pv. *tomato* DC3000 (Niu et al., 2011). In *A. thaliana*, volatile compounds acetoin and 2,3-butanediol produced by *B. subtilis* GB03 elicited ISR dependent on the ET signaling pathway (Ryu et al., 2004; Yi et al., 2016).

Entry of a pathogen into host tissue is a critical early step in infection. For foliar plant pathogens, natural surface openings, such as stomata, are important entry sites (Melotto et al., 2008). Melotto et al. (2006) and Kumar et al. (2012) have shown that the foliar bacterial pathogen *P. syringae* pv. *tomato* DC3000 can actively enter plant tissues through stomata, and that root colonization by the rhizobacterial species *B. subtilis* FB17 triggered the abscisic acid (ABA) and SA signaling pathways to restrict the stomatal-mediated pathogen entry of DC3000 in *A. thaliana*.

*Phytophthora nicotianae*, an oomycete, is a soil-borne, hemibiotrophic pathogen that infects 72 genera of (predominately solanaceous) plants (Hickman, 1958). In tobacco, it can cause black shank disease, symptoms of which include leaf wilting, root rot, stem blackening, and death (Scharte et al., 2005). Several studies have demonstrated that the hyphae of *Phytophthora* can enter the plant leaf via the stomata to initiate disease progression, and the fraction of open stomata strongly decreased after the early stage of *P. nicotianae* infection but reopened again at later stages of the infection that are associated with pathogen entry (Scharte et al., 2005; Hardham, 2007; Zhang et al., 2012).

*Bacillus amyloliquefaciens* FZB42 is a Gram-positive bacterium and a model for the study of plant-microbe interactions. This species is used commercially as a biofertilizer and a biocontrol agent (Chen et al., 2007). Treatment of plants with FZB42 enhances expression of defense genes involved in SA and ET pathways, and it reduces bottom rot caused by *Rhizoctonia solani* in lettuce (Chowdhury et al., 2015). The aims of the present work were: (1) to assess FZB42-induced protection against leaf disease in *N. benthamiana* caused by *P. nicotianae*; (2) to investigate FZB42-mediated stomatal closure and to explore the genetic mechanism; and (3) to identify the signaling pathways involved in plant defenses against *P. nicotianae*.

## Materials and Methods

### Plants and Microorganisms

*Nicotiana benthamiana* seeds were surface sterilized for 5 min in 95% (v/v) ethanol, then for a further 5 min in 5% (w/v) NaClO. Seeds were washed thrice with sterile distilled water, then spread evenly on solid Murashige and Skoog medium (Murashige and Skoog, 1962) to germinate. Seedlings were transplanted to pots containing sterile vermiculite and grown for about 6 weeks in a greenhouse (light intensity 200 μE m^-2^ s^-1^, 50–60% relative humidity, 25°C) with a 16/8 h light/dark cycle. The bacterial strain *B. amyloliquefaciens* FZB42 was deposited as strain 10A6 in the culture collection of the *Bacillus* Genetic Stock Center (BGSC; The Ohio State University, Columbus, OH, United States). *P. nicotianae* was cultured at 24°C for 2 days on clarified V8-agar medium (von Broembsen and Deacon, 1996).

### *Phytophthora nicotianae* Infection Assays

A hyphal plug of *P. nicotianae* (7 mm × 7 mm) was fixed on the surface of *N. benthamiana* leaves (Teng et al., 2014), and the samples were kept in the greenhouse conditions described above. Symptoms of disease were recorded after 48 h. The leaves were then placed in 100% ethanol. The rate of resistance was calculated after measurement of the diameter of *P. nicotianae* lesions: inhibition rate = [(diameter of control lesions-diameter of treatment lesions)/diameter of control lesions] × 100%.

### *Bacillus amyloliquefaciens* FZB42 Root Inoculation

The rhizobacterial strain *B. amyloliquefaciens* FZB42 was maintained on Luria-Bertani medium plates. A single colony from a freshly streaked plate was used to grow overnight cultures that were adjusted to a final density of OD_600_ = 0.5 (10^6^ colony forming units/mL). Root inoculation of FZB42 was performed by pipetting 5 ml of the bacterial suspension onto the roots of 6-week-old *N. benthamiana* plants. Controls were root-inoculated with sterile distilled water. To assess the effect of FZB42 and *P. nicotianae* on stomata, *N. benthamiana* plants were co-inoculated concurrently by FZB42 on the roots and *P. nicotianae* on the leaves.

### Stomatal Aperture Measurements

Stomatal apertures in leaves of *N. benthamiana* plants inoculated with *P. nicotianae*, *B. amyloliquefaciens* FZB42, or co-inoculated with both were measured as described by Kumar et al. (2012). Images of stomatal apertures on epidermal strips were recorded (Olympus BX43 microscope [Tokyo, Japan] and cellSens Standard Software). At least 50 randomly selected stomatal apertures were measured in each treatment, and each assay was repeated three times.

### Plant Hormone Content Determination

To determine ABA content (Fotopoulos et al., 2008), 1 g of freeze-dried, homogenized leaf tissue was extracted in 80% (v/v) methanol and stirred overnight at 4°C. After centrifuging twice at 4000 ×*g* for 20 min, the extracts were completely evaporated under vacuum and dissolved in water at pH 3.0. The solutions were partitioned with diethyl ether three times and then passed through anhydrous sodium sulfate. After evaporation of the apolar phase, the dry residue was dissolved in Tris-buffered saline (150 mM NaCl, 1 mM MgCl_2_, 50 mM Tris–HCl, pH 7.8), and the ABA was detected immunologically using a Phytodetek Kit (Agdia, Elkhart, IN, United States).

Free and SA-conjugated phytohormones were extracted from flash-frozen leaf tissue (1 g) and quantified according to Schuhegger et al. (2006). SA was detected using a Shimadzu RF 535 fluorescence detector at excitation and emission wavelengths of 305 and 407 nm, respectively.

For JA determination, leaves were flash-frozen in liquid nitrogen and tissue (1 g) was processed as described by Mueller and Brodschelm (1994). JA was quantified by gas chromatography-mass spectrometry (GC-MS; SSQ quadrupole instrument; Finnigan, United States) in negative ion chemical ionization mode with isobutane as the reactant gas. Dihydro JA-pentafluorobenzyl (PFB; m/z = 211) and [molecular anions-PFB]-ions of JA-PFB (m/z = 209) were monitored. JA levels were calculated from the GC peak areas of the selected ions.

The concentration of ET was determined from flash-frozen leaf tissue (1 g) according to the method of De Laat and Van Loon (1983).

### RNA Isolation, RT-PCR, and qRT-PCR

Total RNA was extracted from leaves of *N. benthamiana* according to the method of Wu et al. (2017) using a Plant RNA Kit (Omega Bio-Tek, United States). First-strand cDNA was synthesized using reverse transcriptase (TaKaRa Bio Inc., Dalian, China) and oligo(dT) primers. Reverse transcription PCR (RT-PCR) products were examined by agarose gel electrophoresis. Quantitative RT-PCR (qRT-PCR) was performed using SYBR Premix Ex *Taq* (TaKaRa) and an ABI 7500 Fast Real-Time PCR Detection System (Applied Biosystems, United States). PCR reactions were heated to 95°C for 3 min, followed by 30 cycles of 95°C for 30 s, 60°C for 30 s, and 60°C for 30 s. Gene expression in each sample was normalized to expression of *N. benthamiana EF-1α*, and relative expression levels calculated by the 2^-ΔΔ*C*^_T_ method (Livak and Schmittgen, 2001). Gene-specific PCR primers shown in **Table 1** are same for RT-PCR and qRT-PCR analysis.

**Table 1:** Oligonucleotide primers used for gene-specific amplification in this study.

Name	Sequence (5′–3′)		
**RT-PCR and qRT-PCR analysis**			
EF1α-F	ATGATTACTGGTACCTCCCG	EF1α-R	ACCTAGCCTTGGAATACTTG
NCDE-F	CGACCCACGAGTCCAGATTTC	NCDE-R	GAGCCTAGCAATTCCCGAGTG
ICS1-F	CTATCAACGGTGCCATCT	ICS1-R	ATTCCAGCGACACTAACT
PR1a-F	CGTTGAGATGTGGGTCAATG	PR1a-R	CCTAGCACATCCAACACGAA
LOX-F	CCTTAAGAGGAGATGGAACT	LOX-R	TCTAAGCTCATAAGCAATGG
ERF1-F	GCTCTTAACGTCGGATGGTC	ERF1-R	AGCCAAACCCTAGCTCCATT

### Statistical Analysis

At least five replicates were performed for each experiment. Data were evaluated using one-way analysis of variance and Fisher’s least significant difference tests in SPSS software v. 16.0 (Chicago, IL, United States).

## Results

### *B. amyloliquefaciens* FZB42 Treatment Induced Resistance to *P. nicotianae* Infection in *N. benthamiana* Plants

**FIGURE 1:**
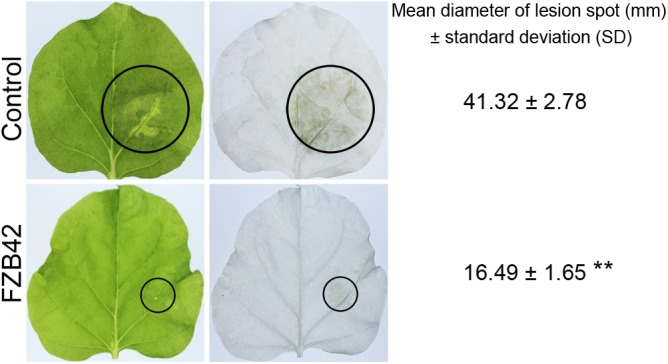
Root-associated *Bacillus amyloliquefaciens* FZB42 induces resistance against the foliar pathogen *Phytophthora nicotianae* in *Nicotiana benthamiana*. Photographs of the lesions were taken 48 h post-inoculation, and the lesion diameters (black circles) were measured. Both the original and ethanol-bleached images are shown. ^∗∗^Highly significant difference compared with the control (*P* < 0.01, *n* ≥ 5).

*Bacillus amyloliquefaciens* FZB42 was tested for its capacity to trigger resistance to the oomycete pathogen *P. nicotianae*. *N. benthamiana* roots were inoculated with a suspension of *B. amyloliquefaciens* FZB42 cells. Leaves were inoculated with *P. nicotianae*. Disease symptoms were assessed 48 h after *P. nicotianae* infection by comparing the sizes of the lesions. Plants inoculated with *P. nicotianae* showed typical symptoms of *Phytophthora* infection; the leaves were water-soaked at 48 h post-inoculation. However, inoculation of roots with *B. amyloliquefaciens* FZB42 resulted in a significant reduction (*P* < 0.01, *n* ≥ 5) in the lesion size compared with control plants treated with *P. nicotianae* alone; the inhibition rate of FZB42 in controlling the leaf disease caused by *P. nicotianae* was 60.09% (**Figure 1**), which suggests that pretreatment with FZB42 can provide enhanced resistance to *P. nicotianae.*

### FZB42 Treatment Caused Stomatal Closure in *N. benthamiana*

**FIGURE 2:**
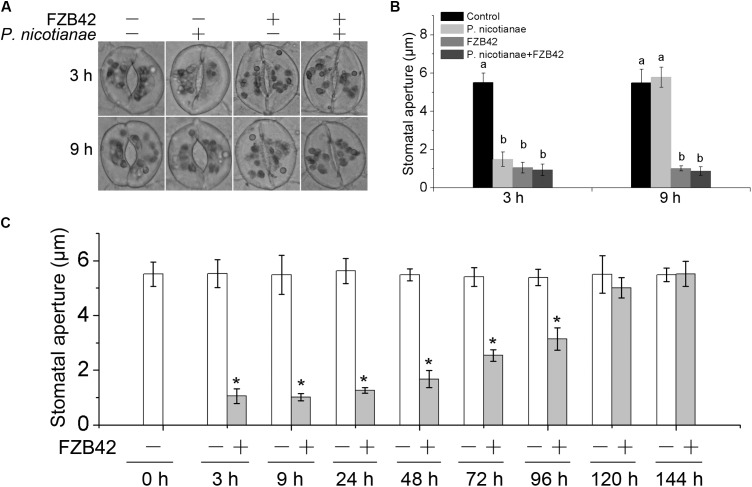
Root inoculation with *B. amyloliquefaciens* FZB42 causes stomatal closure in *N. benthamiana.* **(A)** Micrographs of stomata in leaves of *N. benthamiana* inoculated singly with *P. nicotianae*, *B. amyloliquefaciens* FZB42, or both (co-inoculated). **(B)** Stomatal aperture sizes in leaves of *N. benthamiana* inoculated with *P. nicotianae*, FZB42, or co-inoculated, at 3 and 9 h post-inoculation. Different letters indicate that the means are statistically significantly different at *P* < 0.05. **(C)** Stomatal aperture sizes in *N. benthamiana* after FZB42 root inoculation measured over a time period of 144 h. Data represent means ± standard deviation (SD) in **B**,**C**. ^∗^Significant difference compared with the control (*P* < 0.05; *n* = 50).

Previous studies have shown that root-associated *B. subtilis* restricts the stomate-mediated entry of the foliar pathogen *P. syringae* pv. *tomato* DC3000 in *A. thaliana* (Kumar et al., 2012). To investigate whether stomatal closure contributes to FZB42-mediated defense, roots of *N. benthamiana* were inoculated with FZB42 and stomatal apertures were subsequently measured by microscopic evaluation of freshly prepared epidermal peels. We observed that root inoculation with *B. amyloliquefaciens* FZB42 resulted in a decrease in the mean size of the stomatal aperture 3 and 9 h post-inoculation, while stomata in the control leaves remained open over the entire investigation period (**Figures 2A,B**). *P. nicotianae* triggered stomatal closure 3 h after inoculation, and that the stomata reopened by 9 h post-inoculation (**Figures 2A,B**). Because both FZB42 and *P. nicotianae* can influence the stomata, stomatal apertures were evaluated after co-inoculating *N. benthamiana* plants with FZB42 on the roots and *P. nicotianae* on the leaves. At 3 h post-inoculation, FZB42 and *P. nicotianae* had no significant difference on reducing the stomatal apertures compared with either FZB42 or *P. nicotianae* inoculation alone. Surprisingly, at 9 h post co-inoculation, *B. amyloliquefaciens* FZB42 prevented the reopening of stomata by disrupting the effect of *P. nicotianae* infection (**Figures 2A,B**).

Since FZB42 treatment can specifically keep stomata closed, we root-inoculated *N. benthamiana* plants with FZB42 and examined the sizes of stomatal apertures over a 144-h time course. Interestingly, we found that closure of stomata induced by inoculation of roots with *B. amyloliquefaciens* FZB42 was only a transient response by the plant. The stomatal aperture sizes decreased to ∼1 μm by 3 h after inoculation with strain FZB42, but then there was a gradual increase in the stomatal aperture sizes starting around 24 h (**Figure 2C**). At 120 h post-inoculation, the sizes of the stomatal apertures were similar to those observed in the control. These data indicated that FZB42-mediated stomatal closure in *N. benthamiana* was transient.

### ABA and SA Are Required for FZB42-Mediated Stomatal Closure

**FIGURE 3:**
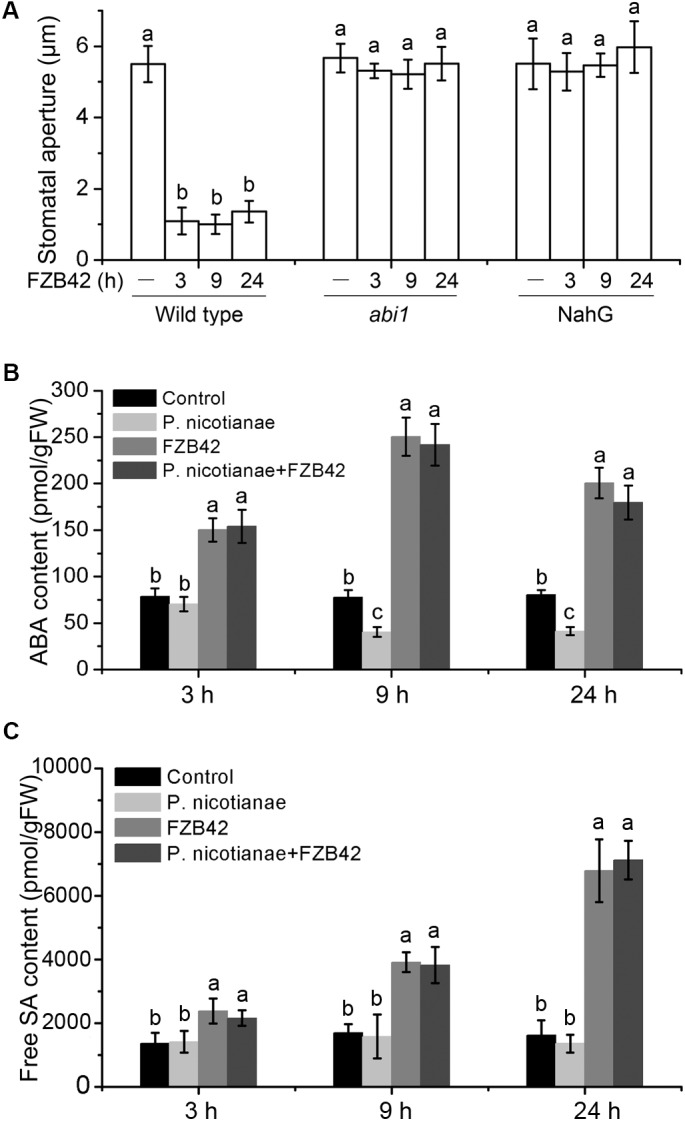
*B. amyloliquefaciens* FZB42 mediates the closure of stomata through abscisic acid (ABA) and salicylic acid (SA) pathways. **(A)** Stomatal aperture sizes in wild-type *N. benthamiana*, and transgenic *abi1* and NahG plants after inoculation of roots with *B. amyloliquefaciens* FZB42. **(B)** Total ABA content in *N. benthamiana* leaves inoculated with *P. nicotianae*, FZB42, or co-inoculated, 3, 9, and 24 h post-inoculation. **(C)** Content of free SA in *N. benthamiana* leaves after inoculation with *P. nicotianae*, FZB42, or both, 3, 9, and 24 h post-inoculation. Data are means ± SD. Different letters indicate significant differences (*P* < 0.05; *n* ≥ 5). FW, fresh weight.

Abscisic acid plays a major role in closure of stomata in response to water stress and pathogen challenge (Acharya and Assmann, 2009; Sawinski et al., 2013; Su et al., 2017). Therefore, to investigate the involvement of ABA in *B. amyloliquefaciens* FZB42-mediated closure of stomata, we measured the stomatal aperture sizes in *N. benthamiana abi1* plants that have a dominant negative mutation in a phosphatase, which impairs ABA transduction during stomatal regulation (Leyman et al., 2000). In the *abi1* mutants, FZB42-mediated closure of stomata was disrupted 3, 9, and 24 h after the addition of *B. amyloliquefaciens* (**Figure 3A**); thus, ABA is required for FZB42-induced stomatal closure. Simultaneously, we measured the mRNA levels of the ABA biosynthetic gene *nced1*, which encodes a 9-cis-epoxycarotenoid dioxygenase that catalyzes the key step in ABA biosynthesis (Tan et al., 1997), and the ABA contents in the leaves of wild type plants at 3, 9, and 24 h after root inoculation with FZB42. There was a significant increase in ABA content (**Figure 3B**), and an increase in transcription of the *nced1* gene (Supplementary Figure S1) compared to non-inoculated control. Our data also showed a marked reduction in ABA content in *N. benthamiana* infected by *P. nicotianae*, while the ABA content remained high during co-inoculation with *P. nicotianae* and FZB42 (**Figure 3B**).

Salicylic acid signaling acts upstream of ABA signaling in *B. subtilis*-triggered stomatal closure in *A. thaliana* (Zeng and He, 2010; Kumar et al., 2012). Therefore, we determined if SA is also involved in FZB42-mediated closure of stomata in *N. benthamiana*. To achieve this, after root inoculation with FZB42 we measured the stomatal apertures in transgenic plants expressing the bacterial *nahG* gene; these plants do not accumulate SA. NahG plants showed disrupted FZB42-mediated stomatal closure 3, 9, and 24 h after root inoculation (**Figure 3A**). We also measured the levels of free and conjugated SA in wild type leaves, and the transcriptional levels of the *ICS1* gene (which is involved in biosynthesis of SA) (Zhu et al., 2014). The SA content was significantly elevated (**Figure 3C** and Supplementary Figure S2), and *ICS1* expression was upregulated after FZB42 treatment compared to control or *P. nicotianae* treatment (Supplementary Figure S3). Collectively, these findings indicate that SA and ABA are both involved in FZB42-mediated stomatal closure in response to *P. nicotianae*.

### The SA-and JA/ET-Dependent Signaling Pathways Are Important in Plant Defenses Against *P. nicotianae*

**FIGURE 4:**
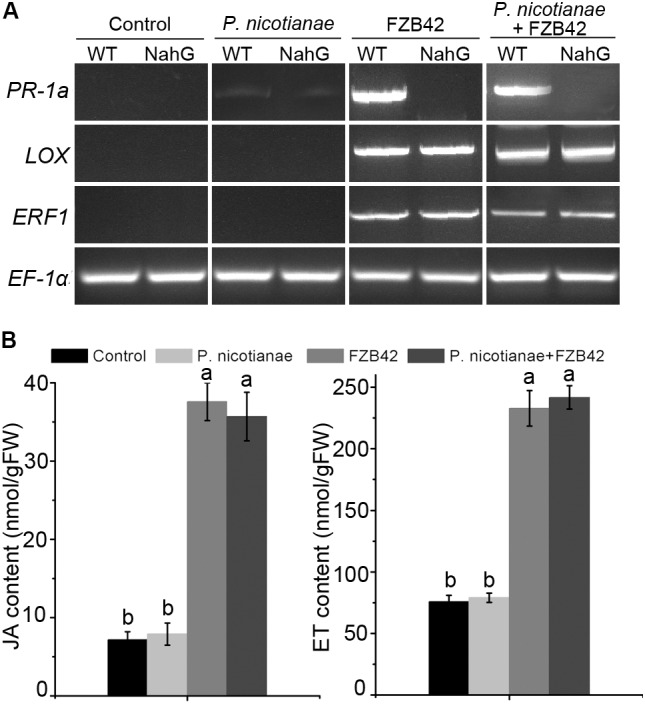
The SA-and jasmonic acid (JA)/ethylene (ET)-dependent signaling pathways are important in plant defenses against *P. nicotianae* in *N. benthamiana*. **(A)** Expression of the *PR-1a*, *LOX*, and *ERF1* genes in *N. benthamiana* wild-type and NahG plants after inoculation with *P. nicotianae*, FZB42, or co-inoculation with both. **(B)** Levels of JA and ET in *N. benthamiana* leaves after inoculation of roots with *B. amyloliquefaciens* FZB42. Different letters indicate significant differences at *P* < 0.05. FW, fresh weight.

To investigate the potential signal transduction pathways involved in FZB42-mediated resistance, we analyzed the expression of the SA-responsive gene *PR-1a*, the JA synthesis-related gene *LOX*, and the ET-responsive gene *ERF1* in the leaves of wild type *N. benthamiana* plants in response to FZB42 treatment alone, to *P. nicotianae* inoculation alone, and to FZB42 treatment combined with *P. nicotianae* inoculation. At 24 h following treatment with FZB42, expression of the *PR-1a*, *LOX* and *ERF1* genes was evident in wild type plants (**Figure 4A**). We observed similar gene expression patterns in *N. benthamiana* plants treated with *B. amyloliquefaciens* FZB42 then challenged with *P. nicotianae*. In plants inoculated with *P. nicotianae* alone, expression of the three marker genes was either very low or not detectable (**Figure 4A**). Results from transgenic NahG, JA signaling-related gene *COI1*-silenced and ET signaling-related gene *EIN2*-silenced *N. benthamiana* plants (Shibata et al., 2010) also have stated this (**Figure 4A** and Supplementary Figure S4). qRT-RCR analysis also showed that transcription of the *PR-1a*, *LOX*, and *ERF1* genes was significantly upregulated after treatment with FZB42 and *P. nicotianae* (Supplementary Figure S5). Meanwhile, after treatment with *B. amyloliquefaciens* FZB42, the free and conjugated SA contents were elevated between four- and sixfold (**Figure 3C** and Supplementary Figure S2), and the JA and ET contents increased five and three-fold, respectively (**Figure 4B**). On the other hand, reduced resistance of transgenic NahG, *COI1*- and *EIN2*-silenced plants was scored by visible development of disease symptoms and inhibition rate (Supplementary Table S1). These data show that SA-and JA/ET- dependent signaling pathways were important in plant defenses against *P. nicotianae*.

## Discussion

A product based on *B. amyloliquefaciens* FZB42 is commercially available (Chen et al., 2007), and although it is used widely as a biocontrol agent, the complicated mechanisms underlying its actions remain to be elucidated. In this study, we characterized the biocontrol mechanism by investigating the FZB42-mediated resistance to *P. nicotianae* in *N. benthamiana* at the phenotypic, cellular, and molecular levels.

Applying FZB42 to the roots of *N. benthamiana* plants reduced the severity of the disease caused by *P. nicotianae* and inhibited proliferation of the pathogen in the leaves, even though *B. amyloliquefaciens* FZB42 only colonizes the roots. Chowdhury et al. (2015) showed that FZB42 treatment can enhance the defense response in lettuce against the fungal pathogen *R. solani*.

Stomata sense plant pathogens and close in their presence (Melotto et al., 2006). *P. nicotianae* infects leaves via stomatal entry, and the reopening of stomata is associated with an increased pathogen concentration in infected plants (Scharte et al., 2005; Zhang et al., 2012). Here, root colonization by *B. amyloliquefaciens* FZB42 restricted pathogen-mediated stomatal reopening in *N. benthamiana*. Meanwhile, FZB42-mediated stomatal closure was transient.

Abscisic acid and SA play a critical role in closure of stomata (Acharya and Assmann, 2009). Our findings also suggest that both ABA and SA are required for the FZB42-mediated closure of stomata. Similarly, Kumar et al. (2012) showed that in *A. thaliana*, root-inoculation of *B. subtilis* FB17 invokes ABA and SA signaling pathways that close light-adapted stomata. Previous studies have demonstrated that ABA/SA-stimulated reactive oxygen species (ROS) production mediated by NADPH oxidases and a peroxidase-catalyzed reaction, respectively, may lead to elevation of cytosolic Ca^2+^, thereby inducing stomatal closure (Pei et al., 2000; Khokon et al., 2011). Here, we showed using the specific fluorescent dye dihydrorhodamine 123 that a ROS burst was observed in *N. benthamiana* after inoculation of roots with FZB42 (Supplementary Figure S6).

As well as stomatal defense, hormonal signaling pathways were also important in plant defenses against the pathogen. Expression of the marker genes *PR-1a*, *LOX*, and *ERF1* involved in the SA, JA/ET signaling pathways was up-regulated in *N. benthamiana* wild-type plants. The levels of the plant hormones SA, JA, and ET also increased. Simultaneously, transgenic NahG, *COI1*- and *EIN2*-silenced plants showed a significant reduction in resistance against *P. nicotianae.* Hence, our data demonstrated that SA and JA/ET signaling pathways play a crucial role in plant defenses against *P. nicotianae*, which agree with results of Niu et al. (2011) and Chowdhury et al. (2015).

## Conclusion

**FIGURE 5:**
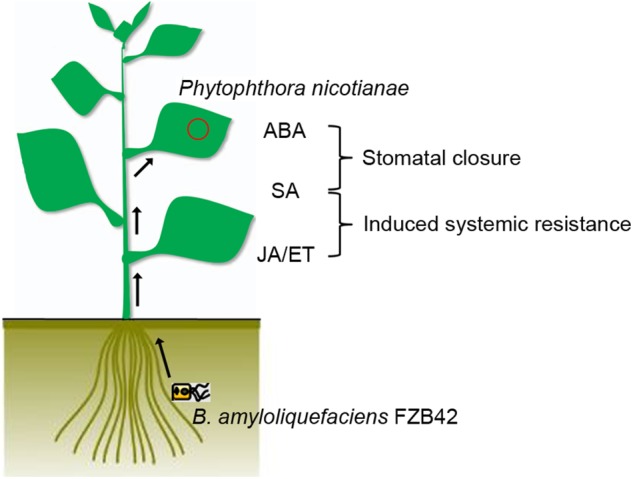
Model of the signal transduction cascade for stomatal defense and hormonal-regulated pathways after FZB42 root inoculation in *N. benthamiana.* After root inoculation with *B. amyloliquefaciens* FZB42, FZB42 mediates guard cell closure through ABA and SA. Meanwhile, concurrent expression of a large set of the SA- and JA/ET-responsive genes in the leaves was activated, suggesting the SA-, JA-, and ET-dependent signaling pathways were also important in plant defenses against *P. nicotianae.*

Stomatal closure and SA, JA/ET signaling pathways are essential for the rhizobacterial species *B. amyloliquefaciens* FZB42 to protect the plant from infection by the foliar pathogen *P. nicotianae* (**Figure 5**). These results provide a deeper understanding of the efficiency of biocontrol agents that affect the entry of a pathogen into its host, although future research has to confirm whether *Bacillus* has similar effect on necrotrophic pathogens.

## Author Contributions

LW, HW, ZW, and XG conceived and designed the experiments. LW and ZH performed of the most experiments. XL, LM, and QG performed the quantitative real time-PCR. RB supplied *B. amyloliquefaciens* strains. LW and JL analyzed data and wrote the manuscript.

## Conflict of Interest Statement

The authors declare that the research was conducted in the absence of any commercial or financial relationships that could be construed as a potential conflict of interest.
