# Supplementary material for: Stomatal Closure and SA-, JA/ET-Signaling Pathways Are Essential for Bacillus amyloliquefaciens FZB42 to Restrict Leaf Disease Caused by Phytophthora nicotianae in Nicotiana benthamiana
Source: Front Microbiol. 2018 Apr 27;9:847. doi: 10.3389/fmicb.2018.00847 (PMC5934478; doi:10.3389/fmicb.2018.00847)
Supplement: Supplementary file 1 [file Presentation_1.PDF]

# Stomatal closure and SA-, JA/ET- signaling pathways are essential for *Bacillus amyloliquefaciens* FZB42 to restrict leaf disease caused by *Phytophthora nicotianae* in *Nicotiana benthamiana*

Liming Wu[1], Ziyang Huang[1], Xi Li[1], Liumin Ma[1], Qin Gu[1], Huijun Wu[1], Jia Liu[2], Rainer Borriss[3,4], Zhen Wu[5*], Xuewen Gao[1*]

[1]College of Plant Protection, Nanjing Agricultural University, Key Laboratory of Monitoring and Management of Crop Disease and Pest Insects, Ministry of Education, Nanjing 210095, China

[2]Chongqing Key Laboratory of Economic Plant Biotechnology, College of Forestry & Life Science, Chongqing University of Arts and Sciences, Yongchuan 402160, China

[3]Nord Reet UG, Greifswald, Germany

[4]Fachgebiet Phytomedizin, Institut für Agrar- und Gartenbauwissenschaften, Humboldt Universität, Berlin, Germany

[5]College of Horticulture, Nanjing Agricultural University

* Corresponding author

Corresponding author:

Xuewen Gao

Email: gaoxw@njau.edu.cn

Telephone/Fax: 86-25-84395268

Zhen Wu

Email: wzh@njau.edu.cn

**Running title:** *Bacillus amyloliquefaciens* restricts leaf disease

**Table S1. Leaves lesion of wild type, transgenic NahG, *COI1*-silenced, *EIN2*-silenced *N. benthamiana* plants inoculated by *P. nicotianae*.**

| **Plants species** | **Mean diameter of lesion spot (mm) ± SD** | **Inhibition rate (%)** |
|---|---|---|
| Wild Type | 16.49 ± 1.65 | 60.09 |
| TRV | 16.97 ± 2.07 | 58.93 |
| Transgenic NahG | 31.87 ± 3.48 | 22.87 |
| *COI1*-silenced | 26.41 ± 0.97 | 36.08 |
| *EIN2*-silenced | 22.15 ± 2.39 | 46.39 |

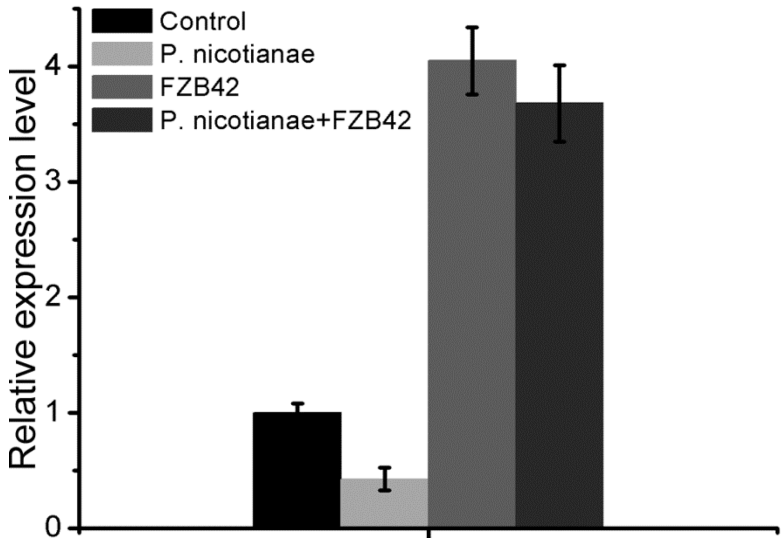


**Figure S1. The mRNA levels of the ABA biosynthetic gene *nced1* in *N. benthamiana* plants after inoculated singly with *P. nicotianae*, *B. amyloliquefaciens* FZB42, or both for 24 h.**

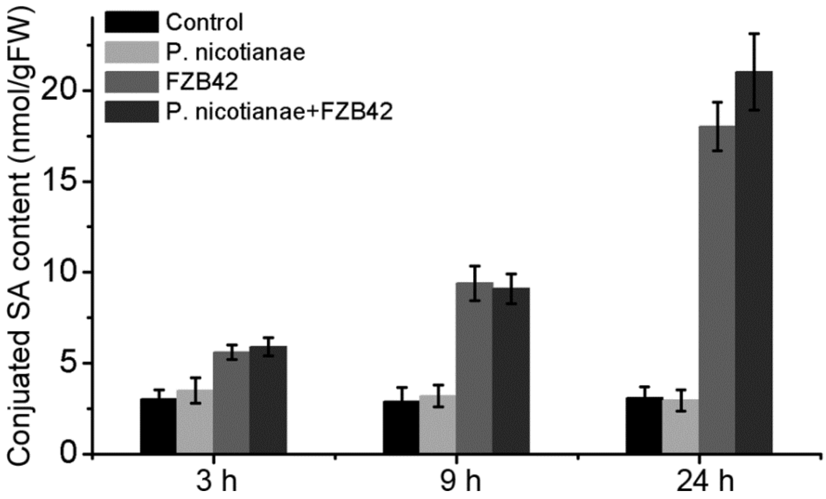


**Figure S2. Contents of conjugated SA in *N. benthamiana* leaves after inoculated singly with *P. nicotianae*, *B. amyloliquefaciens* FZB42, or both.**

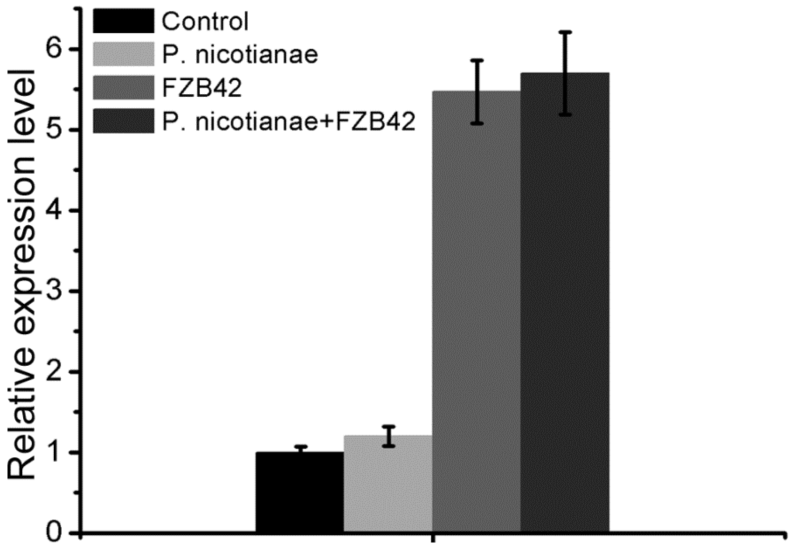


**Figure S3. Quantitative reverse transcription PCR analysis of gene expression for the *ICS1* gene in response to FZB42 inoculation for 24 h.**

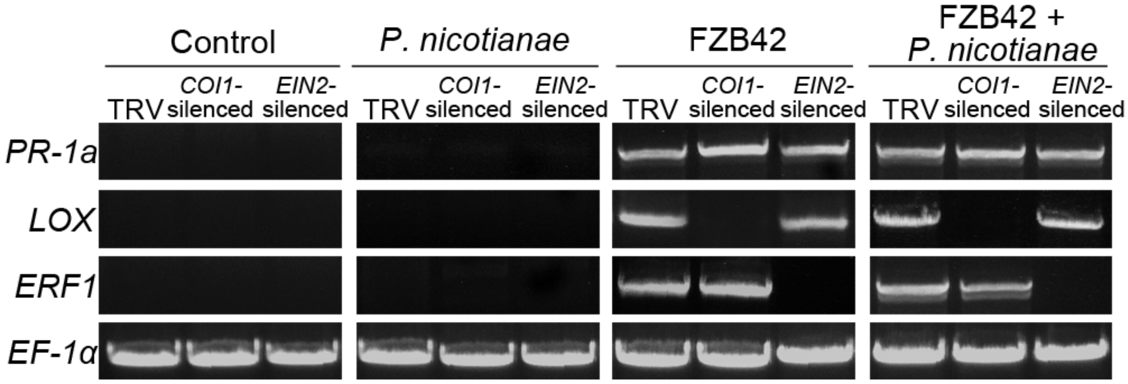


**Figure S4. Expression of the *PR-1a, LOX,* and *ERF1* genes in *COI1-* and *EIN2-*silenced *N. benthamiana* after inoculation with *P. nicotianae*, FZB42, or co-inoculation with both.** *N. benthamiana* was inoculated with *Tobacco rattle virus* (TRV) as the control.

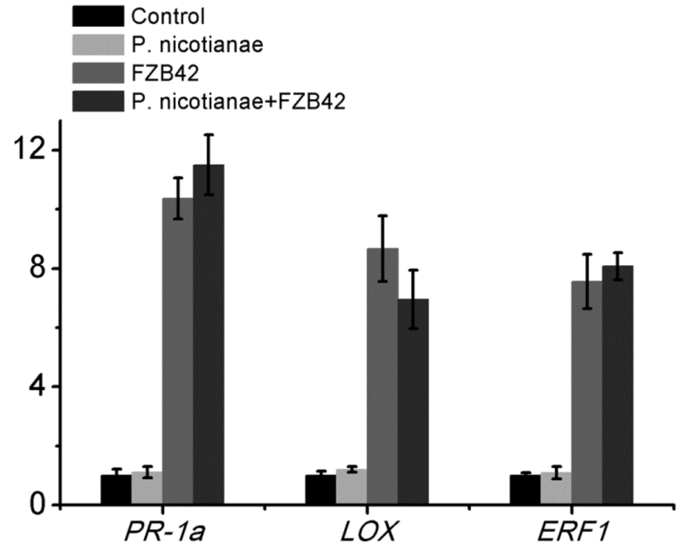


**Figure S5. Quantitative reverse transcription PCR analysis of gene expression for three marker genes in response to *P. nicotianae, B. amyloliquefaciens* FZB42, or both for 24 h.**

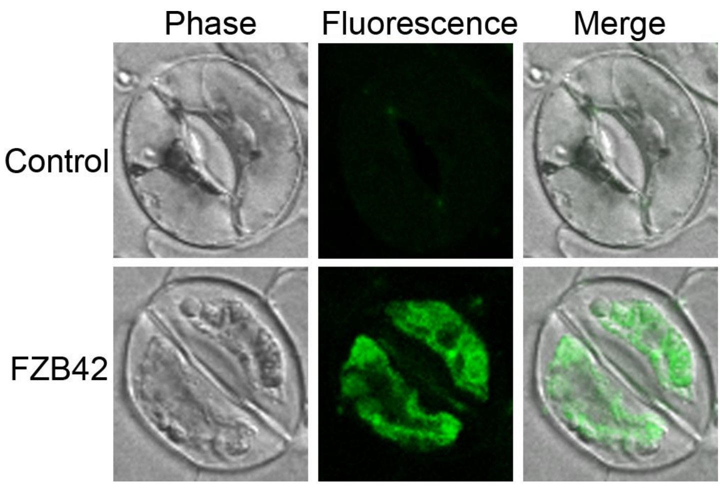


**Figure S6.** ***B. amyloliquefaciens*** **FZB42 root inoculation induced ROS accumulation in guard cells of** ***N. benthamian.***
